# Validation of a new scoring approach of a child dietary questionnaire for use in early childhood among low-income, Latino populations

**DOI:** 10.1186/s40795-022-00618-4

**Published:** 2022-10-31

**Authors:** Laura E. Adams, Evan C. Sommer, Kimberly P. Truesdale, Shari L. Barkin, William J. Heerman

**Affiliations:** 1 Department of Pediatrics, Vanderbilt University Medical Center, 2146 Belcourt Ave, 37232-9225 Nashville, TN USA; 2 Department of Nutrition, University of North Carolina, McGavran-Greenberg Hall, 2209, 27599 Chapel Hill, NC USA

**Keywords:** Nutrition, Childhood obesity, Diet Measurement, Latino families, Diet Quality

## Abstract

**Background:**

Measuring diet quality in early childhood requires time-intensive and costly measurements (e.g., 24-hour diet recall) that are especially burdensome for low-income, minority populations. This study aimed to validate a new method for calculating overall diet quality among low-income, Latino preschoolers.

**Methods:**

This study was an observational study using data from a randomized controlled trial. Participants included parents of Latino preschoolers who reported child diet quality at baseline, 4-month, 7-month, 12-month, and 13-month follow-up. At each timepoint parents responded to a 28-item child dietary questionnaire (CDQ), based on the National Health and Nutrition Examination Survey (NHANES) dietary module, which generated the number of times/day that a child ate each of 28 foods in the past month. These 28 items were then used to create a total standardized child diet quality index (possible range 0-100), using a percent of maximum method. Parents were asked to complete three 24-hour diet recalls at the 13-month follow-up, from which the 2015 Healthy Eating Index (HEI) was derived. Construct validity was evaluated by Spearman’s rank correlations between the new child diet quality index and the 2015 HEI at the 13-month follow-up. Test-retest reliability was assessed by intraclass correlation coefficients (ICC) for sequential pairs of time points.

**Results:**

Among 71 eligible parent-child pairs, mean child age was 4.2 (SD = 0.8) years, 50.7% of children were female, and mean child body mass index (BMI) was 17.8 (SD = 2.0) kg/m^2^. Mean Child Diet Quality Index was 45.2 (SD = 3.2) and mean HEI was 68.4 (SD = 10.5). Child Diet Quality Index and HEI total scores were significantly correlated (r = 0.37; p = 0.001). Test-retest ICCs were statistically significant between all sequential pairs of time points.

**Conclusion:**

The new approach for calculating a measure of overall diet quality from the previously-validated 28-item dietary questionnaire demonstrated modest construct validity. When time and resources are limited, this new measure of overall diet quality may be an appropriate choice among low-income, Latino preschoolers.

**Trial Registration:**

This reports presents observational data collected as a part of a clinical trial, which was registered on clinicaltrials.gov prior to participant enrollment (NCT03141151).

## Background

Dietary quality in children is a primary determinant of life-long health. Yet, the majority of children in the United States do not meet guidelines for dietary quality [1]. These unhealthy patterns of diet quality are established in early childhood, which is a formative period of development where unhealthy nutrition can lead to a lifetime of health consequences [2–4]. The importance of early childhood nutrition is especially relevant for low-income and minority children, as disparities in healthy diet are linked to the development of disparities in obesity, asthma, child development, and educational attainment [5–9].

Despite recognition of the critical importance of early childhood nutrition, there are several limitations to using existing data collection methods to calculate valid measures of child diet quality in minority populations. For example, many existing measures of child diet quality are based on intensive strategies that have high participant burden, often require sophisticated software, and have a high cost to implement. Specifically for minority populations, these existing measures can require a large time burden not only for completing the food record or dietary recall, but for children, can also require them to ask other caregivers to record specific dietary intake if they are not with their child during mealtimes. Marshall, et al. recently conducted a systematic review of available diet quality indices and their associations with health-related outcomes in children and adolescents [8]. They cataloged 80 existing diet quality indices, 44 of which required 24-hour diet recalls, 16 required food records, and 20 used food frequency questionnaires. In addition, these methods are not typically validated in young children or minority populations. Of the 20 food frequency questionnaires identified by Marshall et al., only two of them were studied in children less than 5 years old. These challenges suggest the need for measures of child diet quality that can be calculated from easily administered data collection tools and demonstrate adequate validity in both young children and minority populations.

This leaves researchers with a significant challenge when selecting child diet quality measures and child diet data collection methods that are both appropriate and feasible. While diet quality measures derived from 24-hour diet recalls are typically considered the best available, cost and logistics make them difficult to complete, especially in underserved populations [10]. Additionally, many protocols include completion of 2–3 diet recalls to maximize accuracy of reporting of energy intake [11]. Shortened dietary questionnaires are less burdensome to administer, though they are typically less accurate, less sensitive to issues of serving size and food variety, and more susceptible to bias [12]. These measurement limitations make conducting unbiased and equitable research challenging, both in population-based epidemiological research and in the evaluation of interventions to improve child diet quality. Therefore, developing a method for measuring diet quality in young children that reduces participant burden without significantly compromising validity would be an important tool for conducting equitable research among minority populations.

The purpose of this study was to create and validate a new method for calculating overall diet quality from an existing child dietary questionnaire for use among parents of Latino preschool children from low-income families. Using a cross-sectional study design, diet quality was concurrently measured using two previously validated dietary assessments: (1) a 28-item questionnaire based on the National Health and Nutrition Examination Survey Dietary Screener Module (NHANES), from which our new score was derived, and (2) 24-hour diet recalls, which provide highly reliable estimates of child diet patterns and served as the comparator for the present study. This study tests the hypothesis that the new questionnaire-based diet quality score correlated with the 2015 Health Eating Index (HEI), as derived from 24-hour diet recalls.

## Methods

### Study design

This study used data from the Competency-Based Approaches to Community Health (COACH) randomized controlled trial to prevent and treat childhood obesity and additional diet data collected on a subset of participants immediately following the one-year intervention. Full details of the original study design, intervention description, and results have been previously reported [13, 14]. Though this report does not present the findings from the clinical trial, it was registered on clinicaltrials.gov prior to participant enrollment (NCT03141151). The focus of the current study was to examine the psychometric properties of a new scoring approach to calculating a measure of overall diet quality from a previously validated 28-item child dietary questionnaire. Psychometric properties of this new scoring approach were assessed by (1) test-retest reliability and (2) construct validity by evaluating the overall and subcomponent correlations with a similar diet construct (HEI scores derived from parent-reported 24-hour diet recalls of child diet). All data collection and informed consent were conducted in the participant’s language of choice (exclusively Spanish for this study) by members of the research team who were native Spanish speakers. Adult caregivers provided informed consent on behalf of their children, and children who were at least seven years old also assented. The Vanderbilt University Medical Center Institutional Review Board approved this study. All participants signed written informed consent prior to participation using an enhanced low-literacy approach [15, 16].

### Participants

Parent-child dyads were recruited to participate in the current assessment at the final data collection time point of a one-year randomized controlled trial of the COACH behavioral intervention [13]. To be eligible for the COACH study, families had to self-identify as Latino, children had to be between the ages of 3–5 years old, have a BMI ≥ 50th percentile, and their families had to qualify for government assistance programs such as Women, Infants, and Children (WIC) or Supplemental Nutrition Assistance Program (SNAP) [17]. The choice to exclusively enroll Latino families was based on the disproportionately high rates of childhood obesity among this population and the need for tailored strategies to address this health disparity [18]. While it was not an inclusion criterion to speak Spanish, all of the participants enrolled in the study identified Spanish as their preferred language. Recruitment for the initial study occurred at local community centers, physician waiting rooms, and other community locations where participants were approached by members of the research team to gauge interest and eligibility for the initial study. There were no additional inclusion or exclusion criteria for this dietary validation study, though to be included in the analysis, participants were required to have full data on the child dietary questionnaire and 24-hour diet recall data.

### Child dietary questionnaire

The child dietary questionnaire was based on the NHANES Dietary Screener Module[19] and was collected from parents at baseline and 4, 7, and 12 months after study start, along with other data, including demographics. The exact items used for the current study were previously used in the Infant Feeding Practices Study, which was a nationally distributed consumer opinion panel where a majority of respondents were from relatively high socioeconomic backgrounds [20]. This 28-item child dietary questionnaire asked about the frequency with which children consumed specific food types over the last month, with each item on the questionnaire corresponding to a different food type. The NHANES version of this questionnaire has previously been shown to accurately report mean intake of specific foods (e.g., fruits and vegetables) when validated against 24-hour diet recalls [19]. The way this questionnaire describes diet intake is by measuring food frequency per day for each item, calculated from responses to a two-part question: Part (1) “During the past month, how often did your child eat/drink [food/beverage item]?” Response options were: per day, per week, per month, or do not eat; and Part (2) “How many times?” Response options were integers on the scale indicated in part 1. Individual responses on the per week or per month scale were transformed into number of times per day by dividing by 7 or 30, respectively. For those who opted in, the child dietary questionnaire was collected again at 13 months, along with 24-hour diet recalls. The 13-month child dietary questionnaire was administered immediately following completion of the 24-hour diet recalls, and within 2–4 weeks of the 12-month time point to strengthen comparability of the responses. Items on the child dietary questionnaire were translated into Spanish and back-translated into English by natively bilingual study staff. The questionnaire was delivered by guided administration to account for low-literacy levels in the population. All questionnaire data were entered and stored in REDCap [21].

In previous uses of this 28-item child dietary questionnaire, the items have been used as stand-alone outcome variables without combining them into a single index of diet quality. To create a single composite index of child diet quality from these 28-items, the following method was used: for each of the questionnaire items, frequency of consumption per day could range from zero to an item-specific maximum based on distributional outlier analyses and recommendations from a dietician (e.g., the maximum for the fruit item was five times per day, and the maximum for pizza was one). In a process similar to the aggregation of food items into their respective dietary components for calculation of the HEI-2015, the questionnaire items were categorized into 13 HEI-2015 subcomponents (nine healthy and four unhealthy) (see Table 1) [22]. Note that to accurately represent nutritional composition, some items were categorized into more than one subcomponent (e.g., cereal was categorized as whole grain, refined grain, and added sugar). Within each of the 13 subcomponents, items were summed and standardized using a percent of maximum possible methodology to avoid potential issues with z-score standardization in a longitudinal data context [23]. The percent was then translated into a numeric value such that the resulting 13 subcomponent scores could range from 0 to 100, with higher scores on the healthy subcomponents reflecting higher intake of the corresponding healthy food items (a score of 100 required consumption of the item-specific maximum for every item in the subcomponent), and higher scores on the unhealthy subcomponents reflecting lower intake of the corresponding unhealthy food items (a score of 100 required zero consumption for every item in the subcomponent). Subcomponent scores were then summed and weighted using the weights assigned by the 2015 HEI scoring guidelines (e.g., whole grains were weighted twice as heavily as whole fruits). This weighting procedure was used to create a total standardized child diet quality index which ranged from 0 to 100 with higher scores reflecting higher intake of healthy foods and lower intake of unhealthy foods. Table 1 shows the weights assigned to each sub-component.

**Table 1:** Child dietary questionnaire subcomponents & item categorization

Subcomponent	Weight	CDQ item categorization
*Healthy*		
Total vegetables (11)	5	Bean (3), other vegetable (2), potato (2), tomato (2), vegetable (2)
Greens and beans (5)	5	Bean (3), vegetable (2)
Total fruits (8)	5	Fruit (5), juice (3)
Whole fruits (5)	5	Fruit (5)
Whole grains (10)	10	Bread (3), cereal (2), pasta (1), popcorn (2), rice (2)
Dairy (16)	10	Cheese (3), ice cream (3), milk (4), no sugar dairy (2), other dairy (3), pizza (1)
Total protein foods (10)	5	Bean (3), fish (1), non-processed meat (2), peanut (2), processed meat (2)
Seafood and plant proteins (6)	5	Bean (3), fish (1), peanut (2)
Fatty acids (1)	10	Fish (1)
*Unhealthy*		
Sodium (10)	10	Fries (1), pizza (1), popcorn (2), processed meat (2), snack (2), soda (2)
Refined grains (11)	10	Bread (3), cereal (2), pasta (1), pizza (1), rice (2), snack (2)
Added sugars (21)	10	Cereal (2), other dairy (3), ice cream (3), juice (3), soda (2), sweetened drink (3), sweets (3), tomato (2)
Saturated fats (12)	10	Fries (1), ice cream (3), pizza (1), popcorn (2), snack (2), sweets (3)

Notes: Numbers in brackets represent the maximum raw score for each food item or subcomponent. For each of the questionnaire items, frequency of consumption per day could range from zero to an item-specific maximum based on distributional outlier analyses and recommendations from a dietician (e.g., the maximum for the fruit item was five times per day, and the maximum for pizza was one). The maximum value for each subcomponent is the sum of the maximums for each item contained within it. For example, the greens and beans maximum is 5 (a maximum of 3 from beans, and 2 from vegetables). To standardize the scores, each of these components was then translated into a score of 0-100 using the percent of maximum methodology described in the methods. Weight represents the weight given to each subcomponent in order to calculate the total score as specified in the Healthy Eating Index 2015 scoring guidelines

### 24-hour diet recalls

Diet recalls were collected from participants who opted in to the 13-month time point and were completed over the phone by parent report of child food and drink intake the day before each recall occurred. Participants were invited to complete three dietary recalls to capture day-to-day variability. Recalls were attempted to be completed on non-consecutive days, with at least one weekend day. Nutrition Data System for Research (NDSR) was used to collect and store diet recall data.[24] For each participant, data from multiple recalls were averaged to create the final analytic variables. To be included in the analysis, participants had to complete at least two diet recalls (71 participants had at least two diet recalls, and 67 participants completed all three diet recalls). Using the HEI-2015 guidelines for the simple HEI scoring algorithm method,[22] 13 HEI subcomponent scores and a total HEI score were calculated from the diet recall data. The HEI was designed to reflect the Dietary Guidelines for Americans. It has been validated using data from NHANES for children two years of age and older [25].

### Additional measures

Families also completed a survey at the baseline timepoint of the original trial (i.e., prior to randomization). Demographic items used in the study included child age, child gender, and highest level of parent education. Both parent and child BMI were calculated from prospectively measured height and weight. Weight was measured to the nearest 0.1 kg using calibrated, research, precision-grade scales with a digital read-out (Seca Model 876, 20 kg calibration weight). Height was measured to the nearest 0.1 cm using a free-standing or wall-mounted stadiometer with a moveable headboard (Seca Model 217, calibrated with a wooden yard stick).

### Statistical methods

Descriptive statistics were used to summarize participant baseline characteristics separately for the sample with full CDQ data for the 0, 4, 7, and 12-month time points and the sample with full CDQ and 24-hour diet recall data at the 13-month time points. Test-retest reliability of the child diet quality index was assessed by intraclass correlation coefficients (ICC) and 95% confidence intervals calculated for each sequential pair of time points using single-rating, absolute agreement, 2-way mixed-effects models.[26] Convergent validity was evaluated by Spearman correlations between the corresponding child diet quality index and HEI score for each of the 13 subcomponent and total scores. All analyses were conducted in Stata version 15.1 (StataCorp) [27]. Statistical significance was defined using two-sided tests with α = 0.05.

## Results

Of the 117 parent-child pairs randomized in the original COACH trial, 71 agreed to additional diet data collection and had full CDQ and diet recall data at the 12 and 13-month time points. Mean child age was 4.2 (SD = 0.8) years, 50.7% of children were female, and mean child BMI was 17.8 (SD = 2.0) kg/m^2^ (see Table 2). Mean parent age was 33.4 (SD = 5.8) years with 46.5% completing some high school or less. Baseline characteristics for the sample with full CDQ data at the 0, 4, 7, and 12-month time points (n = 86) are also presented in Table 2. At 13-month follow-up, mean child diet quality index was 45.2 (SD = 3.2) and mean HEI was 68.4 (SD = 10.5). Test-retest ICCs were statistically significant between all sequential pairs of time points (see Table 3), and the ICC point estimate between 12 and 13 months indicated moderate reliability (≥ 0.50). The standardized child diet quality index and HEI total scores were significantly correlated (r = 0.37; p = 0.001), as were 7 of the 13 subcomponents (see Table 4).

**Table 2:** Baseline participant characteristics^a^

	COACH sample	Diet recall sample
	n = 86	n = 71
**Child Characteristics**		
Gender		
Male	42 (48.8%)	35 (49.3%)
Female	44 (51.2%)	36 (50.7%)
Age at anthropometry collection (years)	4.1 (0.8)	4.2 (0.8)
BMI (kg/m²)	18.2 (2.9)	17.8 (2.0)
BMI percentile	85.1 (14.3)	84.4 (14.0)
**Parent Characteristics**		
Age (years)	32.8 (6.1)	33.4 (5.8)
BMI (kg/m²)	31.2 (6.2)	31.5 (6.3)
Education		
Some high school or less	44 (51.2%)	33 (46.5%)
High school graduate or more	42 (48.8%)	38 (53.5%)

Data are presented as N (%) for categorical variables or mean (SD) for continuous variables. ^a^ The COACH sample consists of participants with full diet data at the 0, 4, 6, and 12-month time points. The diet recall sample consists of participants with full diet data at the 12, and 13-month time points. Limited data was collected at 13 months for diet validation.

**Table 3:** Test Re-Test Reliability. Intraclass Correlations of the child diet quality index between sequential time points

Time points	ICC	95% CI	P value
0–4 (n = 86)	0.52	0.34–0.66	< 0.001
4–6 (n = 86)	0.48	0.31–0.63	< 0.001
6–12 (n = 86)	0.58	0.42–0.70	< 0.001
12–13 (n = 71)^a^	0.50	0.30–0.65	< 0.001

^a^ Limited data was collected at 13 months for diet validation

**Table 4:** Diet measures summary and validity at 13-month time point (n = 71)

	Spearman correlation^a^	P value	Child Diet Quality Index^b^	HEI^b^
Totals: Child Diet Quality Index & HEI	0.37	0.001	45.0 (42.9, 47.1)	68.6 (61.4, 76.2)
Subcomponents: Child Diet Quality Index & HEI				
Total vegetables	0.43	< 0.001	11.7 (6.8, 16.9)	2.3 (1.5, 3.5)
Greens and Beans	0.47	< 0.001	11.4 (5.7, 20.0)	3.3 (0.2, 5.0)
Total fruits	0.32	0.006	25.0 (12.5, 30.4)	5.0 (3.7, 5.0)
Whole fruits	0.31	0.009	20.0 (20.0, 40.0)	5.0 (3.7, 5.0)
Whole grains	0.12	0.326	17.1 (12.9, 21.4)	7.5 (4.5, 10.0)
Dairy	0.43	0.000	16.3 (10.9, 21.2)	10.0 (8.1, 10.0)
Total protein foods	-0.06	0.611	12.3 (9.2, 17.1)	5.0 (4.4, 5.0)
Seafood and plant proteins	0.22	0.067	10.1 (7.1, 16.7)	4.1 (1.1, 5.0)
Fatty acids	-0.09	0.461	14.3 (0.0, 14.3)	2.5 (0.8, 5.2)
Sodium	0.05	0.660	91.9 (89.0, 95.8)	6.6 (4.2, 8.4)
Refined grains	0.16	0.182	83.8 (77.9, 87.1)	7.2 (4.2, 9.3)
Added sugars	0.38	0.001	85.7 (81.6, 90.5)	8.5 (6.3, 10.0)
Saturated fats	0.28	0.017	91.5 (86.9, 95.1)	8.1 (6.6, 9.8)

^a^ Values are Spearman’s rank correlations between the total child diet quality index and the Healthy Eating Index, as well as between each subcomponent of both scores

^b^ Values are median (Q1, Q3) for the total child diet quality index and Healthy Eating Index totals and subcomponents

## Discussion

The newly developed scoring methodology for the modified NHANES child dietary screener produced a child diet quality index that had statistically significant correlations with child HEI scores that were derived from 24-hour diet recalls. The standardized child diet quality index also had moderate test-retest reliability. In addition, the majority of the subcomponents of both the child diet quality index and HEI demonstrated statistically significant correlations. These data should not be taken as evidence that a shortened dietary screener could completely replace 24-hour diet recalls. However, when participant burden, cost, or other logistical considerations preclude the use of a more comprehensive dietary measure, the results from this study support the idea that the standardized scores produced from this methodology may be sufficiently reliable and valid in this population to consider using in both population-based and intervention studies.

The correlation between the standardized child diet quality index and the HEI calculated from 24-hour diet recalls was 0.37. This degree of correlation is consistent with previous studies that have validated scoring approaches to shortened dietary questionnaires in other study populations [28–32]. Of the 13 subcomponents of the CDQ, 6 were not significantly correlated with the HEI subcomponent scores, including whole grains, total proteins, seafood and plant proteins, fatty acids, sodium and refined grains. This is likely because the shortened dietary questionnaire does not adequately measure relevant foods from those domains, suggesting opportunities for future research to improve validity. This may be especially important when considering the range of foods eaten by people from a range of cultures, which are not adequately measured in the current measure. For example, in the current study, one of the factors driving the correlation with 24-hour diet recalls may be that foods commonly eaten by the included population of self-identified Latino families may not be captured on the shortened dietary questionnaire. Two potential areas of future research would be to focus on adding specific foods to capture all relevant subdomains of diet quality more appropriately and with particular attention to cultural diversity.

To our knowledge, this is the first composite measure of child diet quality that has been validated in Latino preschoolers using a shortened dietary questionnaire. Many of the 28-items included in this shortened child dietary questionnaire have been used in multiple studies, including NHANES, but always as single-items, not combining them into a composite index as a proxy for overall diet quality. In addition, these types of short dietary screeners are more typically used in population-based studies. The current study suggests that this novel scoring methodology may create a composite measure that would be appropriate for use in clinical or community-based research as an intervention evaluation methodology.

In the context of intervention studies, this novel scoring method demonstrated reliability similar to other comparable measures as well as acceptable validity. It also had a distribution that is well-centered around the midpoint of the range, suggesting the average has room to shift up or down (unlikely to suffer floor or ceiling effects). It has intuitive face validity. For example, someone who frequently eats healthy items and avoids unhealthy items will have a better score than someone who does the opposite. The main concerns about its use in this context are related to accuracy and sensitivity, which are likely not as good as more resource-intensive measures based on diet recall data. The number and types of foods in the 28-item screener are in broad categories and issues with recall bias make it difficult to accurately state how frequently each type of food is actually consumed and how well frequency of consumption translates into actual amounts.

This study had several limitations. The sample was restricted to low-income, minority Latino families. While this is an important subgroup of the total population with high rates of childhood obesity, the conclusions from this study should not be generalized to other populations. Test-retest ICC reliability statistics do not account for possible mitigating effects of the intervention between sequential time points. This might be expected to result in underestimation of the true reliability of the standardized child diet quality index. When calculating the composite total score from the 28-item dietary questionnaire, some food items were categorized into more than one subcomponent to accurately represent nutritional composition. This reflects the fact that some foods have complex relationships to diet quality. Because of the abbreviated nature of the dietary questionnaire used in these study, one implication is that a food containing multiple dietary subcomponents could potentially exhibit both a beneficial and detrimental effect on diet quality. The overall effects of these items on an individual’s final score would depend on which subcomponents it contained and how each was weighted. However, the item-specific maximums prevent any single item from having an outsized effect. While a total child diet quality index of 0 or 100 should be theoretically possible under the percent of maximum possible scoring methodology, this non-mutually exclusive categorization makes these extreme values unlikely or impossible. However, at least in the current study, the implications of this limitation are likely mitigated by the observed concentration of scores around the middle of the scale, indicating the relative sparsity of such extremely healthy or unhealthy diets.

## Conclusion

In conclusion, the new standardized child diet quality index based on a modified version of the NHANES dietary screener demonstrated high construct validity. The modest correlations between the child diet quality index and the HEI are consistent with other dietary screeners validated in different populations. When time and resources are limited, this new measure of overall diet quality may be an appropriate choice among low-income Latino children.

## Data Availability

The datasets generated and/or analysed during the current study are not publicly available due to limitations of ethical approval involving the patient data and anonymity but are available from the corresponding author on reasonable request.

## Abbreviations

CDQ: Child Dietary Questionnaire
NHANES: National Health and Nutrition Examination Survey
HEI: Healthy Eating Index
ICC: Intraclass Correlation Coefficients
BMI: Body Mass Index
COACH: Competency-Based Approaches to Community Health
WIC: Women, Infants, and Children
SNAP: Supplemental Nutrition Assistance Program
NDSR: Nutrition Data System for Research
